# Orbital corticosteroid injections for the treatment of active thyroid eye disease

**DOI:** 10.3389/fopht.2023.1296092

**Published:** 2024-01-04

**Authors:** Kevin T. Eid, Peter M. Kally, Alon Kahana

**Affiliations:** ^1^ Department of Ophthalmology, Oakland University William Beaumont School of Medicine, Rochester, MI, United States; ^2^ Department of Ophthalmology, University of Utah Moran Eye Center, Salt Lake City, UT, United States; ^3^ Department of Facial Plastic Surgery, Virginia Mason Franciscan Health, Seattle, WA, United States; ^4^ Department of Ophthalmology, William Beaumont Hospital, Royal Oak, MI, United States; ^5^ Kahana Oculoplastic and Orbital Surgery, Ann Arbor, MI, United States

**Keywords:** graves orbitopathy, corticosteroid, orbital, thyroid, Kenalog, clinical activity, teprotumumab

## Abstract

**Purpose:**

To study the efficacy of orbital injections of triamcinolone acetonide mixed 1:1 with dexamethasone in the treatment of active thyroid eye disease.

**Methods:**

Patients that received orbital injection(s) of triamcinolone acetonide mixed 1:1 with dexamethasone for thyroid eye disease were included in this retrospective study. Demographic and clinical data were collected from the pre-treatment and 1 month follow up evaluations. Clinical data included subjective pain and diplopia scores, best-corrected visual acuity, Intraocular pressure, extraocular motility, clinical activity score, Hertel exophthalmometry, and upper eyelid margin to reflex distance.

**Results:**

Fifteen patients, 33 orbital injections, were included in the study. The average patient age was 59.2 years (SD ± 13.0) and 89% female. Subjectively, 67% of patients reported improvement of orbital pain and pressure versus 28% stable and 5% worse (p <0.001). Post-procedure clinical activity score decreased from 3.84 to 3.00 (p = 0.0004). There were no significant differences in upper eyelid margin to reflex distance (4.1 ± 1.4 mm vs. 4.3 ± 2.6 mm, p = 0.45), Hertel exophthalmometry (21.7 ± 9.4 mm vs. 21.8 ± 7.6 mm, p = 0.56), or extraocular motility (21% improved vs. 72% stable and 7% worsening, p = 0.50). No steroid-responsive increases in intraocular pressure or injection-related complications were reported.

**Conclusion:**

Orbital steroid injections can successfully reduce symptoms of TED and may be a reliable tool in the treatment of TED as a relatively safe, fast-acting, efficacious treatment option, particularly as a bridge to other therapies.

## Introduction

Thyroid eye disease (TED) is a complication of autoimmune thyroid disorders characterized by inflammation and swelling of the tissues surrounding the eye, resulting in characteristic symptoms such as proptosis, eyelid retraction, diplopia, and foreign object sensation. Severe restrictive strabismus and progression of the disease to compressive optic neuropathy are vision-threatening complications; therefore, the management and mitigation of disfiguring or sight-threatening complications are paramount (1). Intravenous (IV) and oral glucocorticoid treatments have well-established roles as first and second-line treatment of moderate to severe disease with active inflammation in various guidelines such as the European Group on Graves’ Orbitopathy (EUGOGO) and American and European Thyroid Association (2, 3). Despite advancements in medical treatment for TED, including the use of biologics like teprotumumab, targeting inflammation with corticosteroid therapy is still widely used for its relatively rapid response and low cost (4). IV steroid therapy has been shown to be more effective and safer than oral therapy, while oral therapy is still commonly used due to the easier route of administration (5, 6). However due to the innate pharmacology of steroids, both types of systemic therapies have adverse metabolic, hepatic, cardiovascular and musculoskeletal effects requiring close monitoring and tapering (4).

The use of locally administered steroid therapy is widely used in medicine, and has previously been shown as a way to mitigate systemic adverse events when treating various ocular conditions (7). The systemic absorption of local steroid injections has been shown to be minimal (8), and the systemic side-effect profile of these orbital injections are considerably lessened compared to both intravenous and oral corticosteroid therapy (7). In the context of TED, there have been limited studies showing the efficacy of orbital steroid injections. EUGOGO guidelines describe orbital steroid injection as efficacious and a possible treatment when systemic steroid therapy is contraindicated, but have granted the treatment a 2 out of 4 stars recommendation due to the risk of increased intraocular pressure (IOP) and its rare potential side effects such as orbital lipomatosis and retrobulbar hemorrhage (2). While localized steroid therapy is an already existing treatment option for TED, much remains to be discovered regarding its efficacy, safety, and role as a treatment agent among widely-adopted treatment options.

This study describes our subjective and objective retrospective patient outcomes from orbital injections of triamcinolone acetonide (TA) mixed with dexamethasone in the treatment of active thyroid eye disease in patients that are not undergoing concurrent teprotumumab, IV glucocorticoid, or surgical treatments. We hypothesize that this localized treatment will provide patients with a clinically significant reduction in characteristic symptoms of TED while minimizing systemic side effects, adding to the currently limited pool of evidence supporting the use of local steroid injections for the treatment of thyroid eye disease.

## Materials and methods

A retrospective chart review at a single center between July 2020 and January 2022 with Institutional review board (IRB) approval (Beaumont IRB 2021-126) in HIPPA compliant fashion and in accordance with the Declaration of Helsinki. Consecutive patients that received orbital steroid injection(s) for thyroid eye disease (and no concomitant or recent systemic steroid therapy) were included. Injected medication contained a 1:1 mix of TA 40mg/mL (Kenalog-40, Bristol-Myers Squibb Company, Princeton, NJ) and dexamethasone 4 mg/mL, at between 0.5-1.0 mL dose per injection using a 25-gauge 1.5-inch sterile hypodermic needle. The injections were delivered through the inferolateral quadrant, through the fornix, with the patient staring straight ahead, avoiding the muscles. Once placed into the orbit, the needle was slightly wiggled to ensure that the globe does not move, the plunger withdrawn to ensure no intravascular position, and then the steroid injected slowly and with minimal digital pressure into the intraconal space lateral to the inferior rectus. The volume of injection was dependent on the tension of orbit, as any resistance to injection resulted in no further drug administration. All injections were performed at a single center by a single surgeon (AK). Demographic and clinical data were collected from the pre-treatment and the first follow up evaluation no later than 3 months. The average follow-up period was 4 weeks. Clinical data included best-corrected visual acuity (BCVA), IOP (mmHg), extraocular motility (EOM), clinical activity score (CAS), Hertel exophthalmometry, margin to reflex distance (MRD1), and a subjective “better, worse, or stable” evaluation from the patient. Patients were excluded for incomplete data or if actively undergoing concomitant medical (e.g., teprotumumab, glucocorticoid infusions, immunomodulatory therapy), radiotherapy, or surgical (e.g., orbital decompression) intervention around the time of intervention and during the follow-up period. Concomitant interventions were deemed confounding. Subjects without complete exam data were still included as long as there were consistently tracked pre and post-intervention data for multiple study variables. Quantitative comparisons between pre and post-intervention variables were made using a paired two-tailed t-test, and categorical comparisons were made using Fisher’s exact test.

## Results

**Figure 1:**
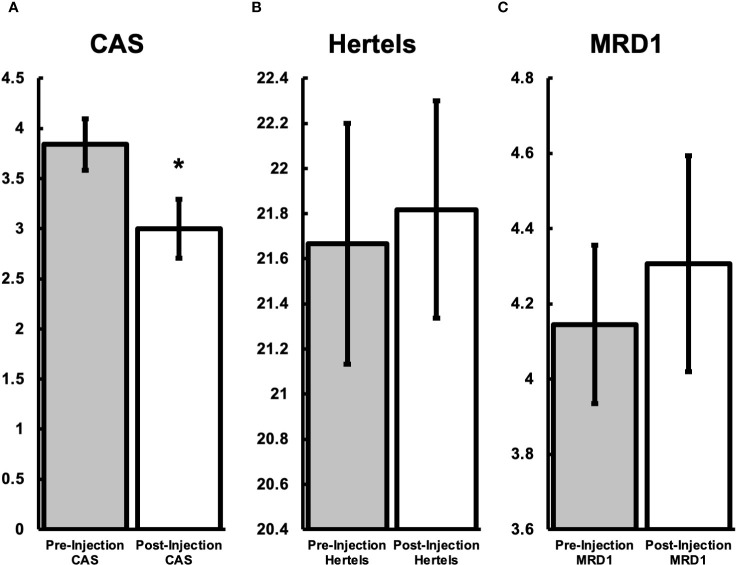
Endpoint comparisons between patients pre-steroid treatment and one-month after treatment. **(A)** Average CAS before the injection was 3.84 (SD ± 1.64). One month after treatment the CAS was 3.00 (SD ± 2.16), a decrease of 0.84 (P<0.01). **(B)** Average Hertel before the injection was 21.7mm (SD ± 3.07). One month after treatment the Hertel was 21.8 (SD ± 2.77), an increase of 0.1 (P=0.56). **(C)** Average MRD1 before the injection was 4.1mm (SD ± 1.4). One month after treatment the MRD1 was 4.3mm (SD ± 2.6), an increase of 0.2 (P=0.45). *Represents statistical significance (P-value <0.05).

Forty-six orbital injections were screened and 33 injections from 15 patients were used in this study. Thirteen injections were excluded for the following reasons: post-intervention data was insufficient (8), missing pre-intervention exam details (4), and lost to follow-up (1). The average patient age was 59.2 years (SD ± 13.0) and 89% female. Average volume of injection was 0.81mL (range 0.5-1.0), with each orbit receiving an average 1.31 injections (range 1-3). The mean pre-intervention average CAS was 3.84 (n=25) (**Figure 1**). The mean post-intervention average CAS was 3.00 (n=25). The ranges of CAS were 2-6 and 1-5 pre- and post-intervention, respectively. The difference between post and pre-intervention CAS was -0.84 (P=0.0004241) (**Figure 1A**). The mean pre-intervention Hertel measurement was 21.667 mm (n=33). The mean post-intervention average Hertel was 21.818 mm (n=33). The difference between post and pre-intervention Hertel was 0.152 mm (P=0.55679) (**Figure 1B**). The mean pre-intervention average MRD1 was 4.145 (n=31). The mean post-intervention MRD1 was 4.306 (n=31). The difference between post and pre-intervention MRD1 was -0.161 (P=0.45482) (**Figure 1C**). Subjective pain and pressure reporting by the patient for general benefit of the injections showed that 67% improved, 28% remained stable, and 5% worsened (p <0.001). For changes in EOM, 21% improved, 72% remained stable, and 7% worsened (p = 0.50).

## Discussion

This study demonstrated that orbital corticosteroid injections can lead to significant improvements in CAS and orbital pain and pressure symptoms at one month post-intervention in patients with TED. The study also showed that after a month post-intervention, there were no significant improvements in EOM, level of eyelid retraction, or level of exophthalmos. We also did not observe a rise in IOP or any significant adverse events during the follow-up visit.

Our study supports the existing literature showing the success of local corticosteroid injections as a reliable treatment option for TED. While our injections targeted CAS symptoms most effectively, a previous study by Lee et al. showed success with reducing upper eyelid retraction using TA injections in the subconjunctival space at the upper margin of the tarsus (9). The study observed an average reduction of MRD1 by 0.98mm (P<0.05) after a two-week evaluation and was seen to be most effective in patients with acute phase thyroid eye disease. Similar findings were observed in Hamed-Azzam et al. where orbital TA injections were performed through the fornix (10). Significant reductions in lid retraction and CAS were seen, in addition to significant reductions in lateral and medial rectus muscle size visualized via ultrasonography. In Ebner et al., computerized tomography was used to demonstrate similar reductions in extraocular muscle size 20 weeks after patients received four weekly doses of orbital TA injections, in addition to observing a significant reduction in extraocular motility areas of diplopia (11).

Similar to our study, previous literature did not demonstrate a significant increase in IOP, or only showed a transient increase in IOP in a small subset of patients that could be managed topically (9–15). A common adverse effect of the treatment in our study population was conjunctival redness for 1-10 days around the injection site that was self-limited. Otherwise, we did not observe any significant adverse events; although, complications, including vascular occlusion, infection, bleeding, and risk of globe rupture, remain a major factor in the medical decision making for particulate steroid-injection into a vascular area (16–18). Additionally, our injections were deep into the orbit, lateral to the inferior rectus muscle, making sure not to inject the muscle because steroids are toxic to muscles and there is a risk of damaging the muscle with an orbital steroid injection (19). We did not observe any intramuscular injections, and there were no signs of hemorrhage or EOM injury following injection. Systemic side effects were also not clinically apparent in our patients. The lack of systemic side effects with orbital steroid injections was also described in Ebner et al. by observing no significant changes in serum glucose, calcium, plasma cortisol, and urinary cortisol after the injections were made (11).

Our study was limited by not having a control group or a larger data set. Comparing pooled data due to some patients having missing data in the follow-up visit also limited the power of the study. There is also expected minor measurement error associated with repeat measuring of Hertel exophthalmometry and MRD1. Given that CAS is a subjective cumulative assessment of thyroid eye disease activity that combines inflammatory and congestive signs and symptoms, we did not deduce what specific improvements are being made after local steroid injection even though improvements in all components of CAS were represented across the study population. Additionally, our study’s follow-up period was limited to the first follow-up (~1-month post-intervention) because patients were exposed to treatments such as teprotumumab or surgery after this time interval thus confounding the outcomes of orbital injections. Most patients’ symptoms reoccurred within 6 months of treatment. Our use of orbital steroid injections was given to symptomatic patients as a short-term, primary mode of therapy that can be perfored immediately in clinic with minimal side effects, acting as a bridge therapy to a more definitive treatment e.g., orbital decompression or teprotumumab. So while these patients have been followed >3 months, endpoint comparisons between pre- and post-injections >1 month could not be made due to confounding variables.

Further study is needed to evaluate onset of action, response longevity, and to compare outcomes to more established treatment options. Comparing different combinations of injected corticosteroids amongst each other is also worth exploration because other studies have used a standalone TA and a mixture of dexamethasone and TA (9–15, 20). In a systematic review by Park and Barmettler, TA was shown to have a higher risk of vascular occlusion related vision loss compared to other local steroid injections, most likely due to its large particle size and extensive particle aggregation (21). There may be decreased risk of vision or sight threatening complications with the use of a combination of dexamethasone and TA than TA alone due to dexamethasone’s higher solubility and decreased particulate density compared to TA (7, 21, 22). Dexamethasone’s higher solubility and higher potency may lead to faster onset, and TA’s poor solubility allows it to remain in the injected area for longer (22). As a result, a mixture of both dexamethasone and TA may be faster acting and longer lasting than either steroid on their own. Previous studies have also shown promising results using a combination of TA and dexamethasone. Bagheri et al. examined patients after four orbital injections of combined TA and dexamethasone and observed a significant decrease in CAS, lid retraction, and exophthalmos (12). Additionally, in Wang et al. regression analysis was used to measure the effect of variables such as disease longevity, disease severity, and number of injections on the effectiveness of orbital injections using TA with dexamethasone (20).

In conclusion, orbital steroid injections can successfully reduce symptoms of TED in non-severe cases and may be a reliable tool in the treatment of TED as a relatively safe, fast-acting, efficacious treatment option, particularly as a bridge to other therapies.

## Data availability statement

The raw data supporting the conclusions of this article will be made available by the authors, without undue reservation.

## Ethics statement

The studies involving humans were approved by Beaumont Institutional Review Board. The studies were conducted in accordance with the local legislation and institutional requirements. The participants provided their written informed consent to participate in this study.

## Author contributions

KE: Writing – original draft, Writing – review & editing. PK: Writing – original draft, Writing – review & editing. AK: Writing – original draft, Writing – review & editing.
